# Prospective Study of One Million Deaths in India: Rationale, Design, and Validation Results

**DOI:** 10.1371/journal.pmed.0030018

**Published:** 2005-12-20

**Authors:** Prabhat Jha, Vendhan Gajalakshmi, Prakash C Gupta, Rajesh Kumar, Prem Mony, Neeraj Dhingra, Richard Peto, RGI-CGHR Prospective Study Collaborators

**Affiliations:** **1**Centre for Global Health Research, Public Health Sciences, St Michael's Hospital, and McLaughlin Centre for Molecular Medicine, University of Toronto, Toronto, Canada; **2**Epidemiological Research Centre, Chennai, India; **3**Healis-Seskarhia Institute of Public Health, Navi Mumbai, India; **4**School of Public Health, Post Graduate Institute of Medical Education and Research, Chandigarh, India; **5**Institute of Population Health and Clinical Research, St. John's Medical College, Bangalore, India; **6**Clinical Trial and Epidemiological Studies Unit, University of Oxford, Oxford, United Kingdom; National Institute of Public HealthMexico

## Abstract

**Background:**

Over 75% of the annual estimated 9.5 million deaths in India occur in the home, and the large majority of these do not have a certified cause. India and other developing countries urgently need reliable quantification of the causes of death. They also need better epidemiological evidence about the relevance of physical (such as blood pressure and obesity), behavioral (such as smoking, alcohol, HIV-1 risk taking, and immunization history), and biological (such as blood lipids and gene polymorphisms) measurements to the development of disease in individuals or disease rates in populations. We report here on the rationale, design, and implementation of the world's largest prospective study of the causes and correlates of mortality.

**Methods and Findings:**

We will monitor nearly 14 million people in 2.4 million nationally representative Indian households (6.3 million people in 1.1 million households in the 1998–2003 sample frame and 7.6 million people in 1.3 million households in the 2004–2014 sample frame) for vital status and, if dead, the causes of death through a well-validated verbal autopsy (VA) instrument. About 300,000 deaths from 1998–2003 and some 700,000 deaths from 2004–2014 are expected; of these about 850,000 will be coded by two physicians to provide causes of death by gender, age, socioeconomic status, and geographical region. Pilot studies will evaluate the addition of physical and biological measurements, specifically dried blood spots.

Preliminary results from over 35,000 deaths suggest that VA can ascertain the leading causes of death, reduce the misclassification of causes, and derive the probable underlying cause of death when it has not been reported. VA yields broad classification of the underlying causes in about 90% of deaths before age 70. In old age, however, the proportion of classifiable deaths is lower. By tracking underlying demographic denominators, the study permits quantification of absolute mortality rates. Household case-control, proportional mortality, and nested case-control methods permit quantification of risk factors.

**Conclusions:**

This study will reliably document not only the underlying cause of child and adult deaths but also key risk factors (behavioral, physical, environmental, and eventually, genetic). It offers a globally replicable model for reliably estimating cause-specific mortality using VA and strengthens India's flagship mortality monitoring system. Despite the misclassification that is still expected, the new cause-of-death data will be substantially better than that available previously.

## Abstract 2

This paper reports the rationale, design, and implementation of the world's largest prospective study of the causes and correlates of mortality.

## Introduction

About 46 million of the estimated 60 million deaths per year worldwide occur in developing countries [1]. However, there is a dearth of reliable and accurate information on the causes and distribution of mortality in these countries. India has about 9.5 million deaths a year, or about one in six of all deaths worldwide. Over three-quarters of deaths in India occur in the home; more than half of these do not have a certified cause.

To meet these modern challenges of mortality measurement, the world's largest prospective study of the causes and correlates of mortality in India is being undertaken by the Registrar General of India (RGI)'s Sample Registration System (SRS). The study, called the Prospective Study of 1 Million Deaths in India, is implemented in close collaboration with the Centre for Global Health Research at the University of Toronto, leading Indian and overseas academic institutions, and the Indian Council of Medical Research. The study has several innovations that are relevant to other developing countries considering the measurement of mortality and to recent calls for improved health statistics [2–5]. It uses a well-validated household instrument to ascertain causes of death and dual recording methods to improve reliability and consistency. It is national in scale, representative of the population, and–by recording underlying demographics–able to quantify absolute mortality rates. Prospective and retrospective study designs, such as household and proportional mortality and nested case-control methods, permit quantification of correlates of mortality.

In this report, we present the rationale, design, results of validation studies to date, key statistical issues, and expansion to biologic measurement for the Million Death Study in India. We discuss the challenges in implementing modern mortality measurement and the implications for global health.

### Rationale: Why Measure Mortality?

Historically reliable, representative, routine, low-cost, and long-term mortality measurements are the key to monitoring trends in health conditions of the population, detecting new epidemics (such as HIV/AIDS), spurring research into avoidable causes of death, evaluating the success of control programs, and improving accountability for expenditures on disease control [6,7]. Routinely collected data have helped to spur further research and public health action and contributed to the enormous increases in life expectancy in the 20th century [8].

Public health in industrialized countries was transformed when vital statistics on age, sex, and socioeconomic distribution of births and deaths became available in the late 19th and 20th centuries. Vital statistics have demonstrated major trends in fertility, child survival, and mortality. They have shown good news, such as the large declines in under-5 mortality and tuberculosis mortality during the 20th century. They have also raised alarm; in the mid-1940s, a dramatic increase in lung cancer deaths in British and American men after World War II led to much research on smoking [9]. In the early 1980s, routine mortality data from San Francisco revealed an exceptional increase in immune-related deaths among young men and signaled the start of the American HIV-1 epidemic [10].

Vital statistics need to keep up with modern patterns of disease. India and other countries have seen consistent decreases in child mortality (under-5 mortality has fallen by about 2% per year since 1971 in India [11]). Adult deaths in middle age (35–69 y) attributable partially to the effects of smoking, sedentary lifestyles, and higher saturated fat intake have been on the rise. More recently, deaths among young adults (15–34 y) have risen from HIV/AIDS. Reliable information on the diseases of adults and their causes are a large gap in global knowledge.

Recorded deaths from most communicable diseases or injuries generally correspond to their causes (for example, malaria deaths are caused by *Plasmodium* parasites), but the more “chronic” communicable diseases (such as tuberculosis) and most noncommunicable diseases can have multiple causes. For example, a myocardial infarction could be caused by smoking, elevated blood pressure, high lipids, or other factors. The age- and sex-specific importance of established risk factors, or combinations of risk factors, has only recently been reliably documented through appropriately large studies in Western populations, and with surprising results. For example, the association of blood pressure and vascular disease is twice as steep as previously believed [12] if blood pressure is measured reliably and the effects of “regression-dilution” bias [13] are properly considered. There are few epidemiological studies that document the age-, sex-, and region-specific hazards of blood pressure, blood lipids, and smoking in developing populations [14], where most of the world's vascular deaths occur [1].

Anecdotal evidence suggests that in India each disease that is common in one part of the country is relatively uncommon elsewhere, for reasons that are not understood. This means that there are important avoidable causes that still await discovery. Much more remains to be discovered about the novel genomic, proteomic, and other biochemical correlates of respiratory, intestinal, or other infections in general, and of the avoidable causes of chronic diseases such as cancer, heart attack, stroke, and lung disease[15,16] that currently account for most of the adult mortality in India. Even for infections such as HIV-1 and tuberculosis, there may well be genetic causes (such as polymorphisms in genes involved in innate [17,18] or adaptive [19] immune recognition) or environmental causes (such as other infections [20,21] other than the relevant pathogens) that make particular infections, or progression from infection to disease, more probable.

### Alternative Designs for Measuring Mortality

The ideal mortality measurement system has several characteristics [7]. It is routine, reproducible, long-term, low-cost, and sustainable. It is reliable and representative of the population (implying that it avoids major selection biases in enrollment). It captures not only the death, but also reliably the cause based on the *International Statistical Classification of Diseases and Related Health Problems* (ICD-10 [22]). The ideal system includes not only events or “numerators,” but also underlying population demographics (“denominators”).

Three systems measure mortality in India (http://www.censusindia.net/). The first, the Civil Registration System, is currently unreliable due to gross underregistration. While some areas have very good vital registration (Mumbai provides death registration as far back as 1848 [23]), overall, only 3.5 million of the estimated 9.5 million annual deaths were registered in India in 1999. Among registered deaths, cause-of-death data are available for about one in three deaths, but this often merely subdivides deaths as due to accident, violence, or disease, without further details. Civil registration is the accepted mortality measurement system in Western countries where coverage nears 100%. However, access to medical care is far less common in India, and most deaths occur at home rather than in hospitals. Eventually, civil registration will increase, as has happened recently in China. But this may take decades; the United States took the better part of a century to increase death certification, and some states did not have complete coverage until the 1970s [24].

The second system is the Medically Certified Causes of Death. However, this covers only about 0.4 million deaths and is largely confined to selected urban settings that are not representative of the general population. Problems with inconsistent physician attribution of causes of death, especially for senility and ill-defined causes have been noted [6,7].

The third is the SRS (described in detail below). Only the SRS is representative of both urban and rural settings of India, covering some 6,700 to 7,600 units randomly selected from the preceding census. The SRS is much smaller, though, covering only 0.05 million deaths. Thus, its chief drawback is that it cannot yet provide district-level data for local decision making, and it lacks sufficient power to generate yearly rates for less common causes, such as maternal deaths. Until recently, the SRS did not adequately capture information on causes of death. However, we have addressed this gap by developing and implementing verbal autopsy (VA)–an innovative method to estimate cause-specific mortality.

Finally, it is worth noting that econometric models of cause-specific mortality [1] are no replacement for direct measurement. Indirect estimates are only as good as their underlying data. The econometric models have not been well tested in the presence of HIV/AIDS growth. In India the global burden of disease varies considerably depending on the assumptions used. For example, the 1994 global burden of disease estimated 0.78 million cancer deaths in 1990, but cancer registry data suggested a much lower figure of 0.43 million deaths [25]. The 1996 version of the global burden of disease projected 0.95 million deaths from tuberculosis in 2000; the 1999 version estimated 0.42 million deaths in 1998 [26].

### Study Objectives

In light of the above, we have the following objectives: (a) to reliably document cause-specific mortality from 2001 to 2003 (3 y; approximately 150,000 deaths) within the SRS to establish regional-, gender-, and age-specific variation and patterns of mortality; (b) to document causes of death with routine use of VA in the new SRS sampling frame from 2004 to 2014 (10 y; approximately 700,000 deaths expected); (c) to improve our understanding of selected risk factors, most notably tobacco use, alcohol use, indoor air pollution, fertility preferences for male children and its effect on female survival, immunization, and migration through linking of mortality outcomes with exposure status, using retrospective and prospective methods; and (d) to expand the new SRS to obtain reliable epidemiological evidence about the relevance of physical (such as blood pressure and peak flow), behavioral (such as migration and HIV-1 risk), and biological (such as blood lipids and gene polymorphisms) measurements to the development of disease in individuals or disease rates in populations.

## Methods

### Study Setting

This study is conducted within the SRS, a large, routine demographic survey and the primary system for the collection of Indian fertility and mortality data since 1971 [27]. There are two SRS sample frames. The first SRS sample frame covers 6.3 million people (including 2.9 million adults aged 25 y or older) in all 28 states and seven union territories of India. An average of 150 households are drawn from each of 6,671 sample units (4,436 rural and 2,235 urban), which in turn are selected using 1991 census data. The new SRS sample frame covers about 7.6 million people (including 3.5 million adults aged 25 y or older) in all 28 states and seven union territories of India. Households are drawn from 7,597 sample units (4,433 rural and 3,164 urban) selected from the 2001 census.

SRS sample units are randomly selected to be representative of the population at the state level. The sample design is a unistage stratified simple random sample without replacement. The sample size for the first and new SRS sample frames are based on total fertility rates and infant mortality rates, respectively. Within the SRS, selected households are continuously monitored for vital events by two independent surveyors. The first is a part-time enumerator (commonly a local school teacher familiar with the area/village) who visits the home every month. The second is a full-time (nonmedical) RGI surveyor who visits the home every 6 mo. Each independently records the births and deaths in the household for a 6-mo period. A third staff member does a reconciliation of the two reports, arriving at a final list of births and deaths for each household, which completes each half-yearly survey. The RGI surveyors each cover about 150 households with a total average population of 900 (ranging from 700 to 1,500), and report about 50 deaths (and about 150 births) every 6 mo.

### Plan of Investigation

**Figure 1:**
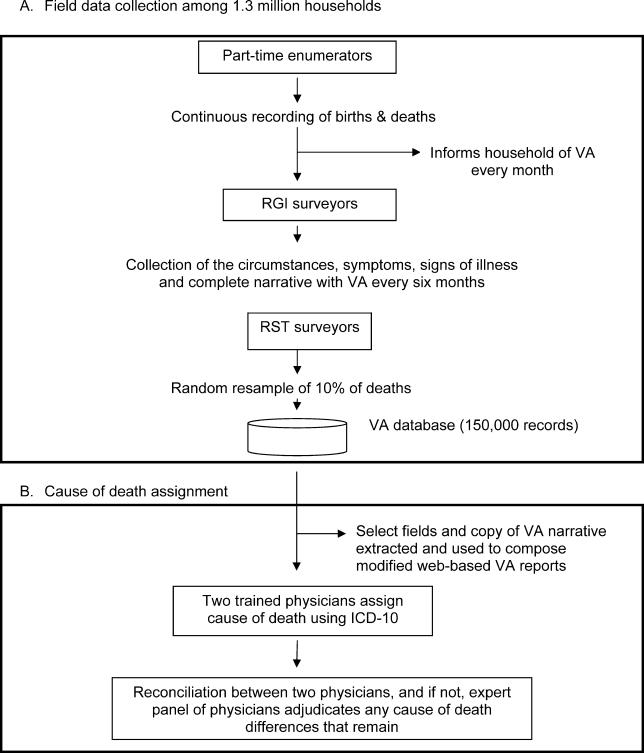
SRS VA Activities

Box 1 and Figure 1 provide an overview of the study methods. In brief, the method involves 800 trained (nonmedical) RGI surveyors implementing VA reports among enrolled populations every 6 mo. A random 10% of the VA fieldwork is repeated by an independent audit team. After data entry, field reports are sent electronically to two independent and trained physicians who assign cause of death, based on the ICD-10 [22], using a Web-based system. The two physicians have to agree on the underlying cause of death, and if they do not, such records undergo reconciliation and third-physician adjudication.

**Box 1. SRS VA Methods Overview**

**Design of verbal autopsy questionnaire.**Combined open/closed format. Structured questions accompanied by an open-ended narrative. Symptom list to assist attribution of deaths.

**Questionnaire layout.**One-page, double-sided, scannable forms. Four age-specific forms (neonatal, child, adult, and maternal). Forms available in either English or Hindi.

**Interviewers.**Nonmedical RGI surveyors (mostly male) with knowledge of local language(s) and trained in VA instrument.

**Interview technique.**One-on-one interviews during home visits. Duration of 30–45 min.

**Respondents.**Family members or other informants (usually neighbors or close associates of the deceased).

**Recall period.**Usually less than 6 mo, but still valid up to 3 y.

**Data quality.**Random resample of 10% of all deaths to ensure completeness of fieldwork.

**Derivation of diagnosis.**Central medical review of cause by two independent physicians using modified VA reports (Physician Reports) and an Internet-based Web application. Adjudication of disagreements by an expert physician.

**Mortality classification.** *The International Classification System of Diseases and Related Health Problems,* Tenth Revision (ICD-10).

**Sample size.**One million deaths (about 0.3 million from 1998–2003, 0.7 million from 2004–2014).

Text S1–S3 provide the details of the field collection methods, resampling, physician coding, data management, and research ethics. The full protocol, field instruments, training manuals, slide presentation of validation results, and procedures are available at http://www.cghr.org/project.htm.

### Estimated Sample Size and Distribution

**Figure 2:**
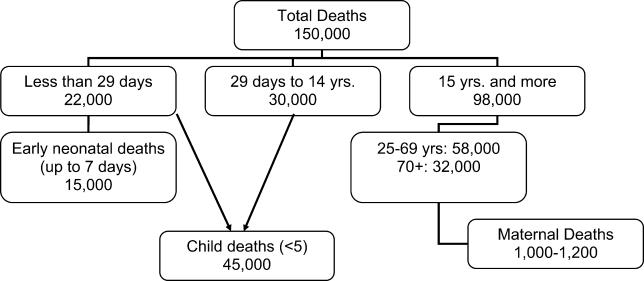
Expected Number of Deaths by Age Group Using VA (Thousands), 2001 to 2003

The primary outcomes of interest are all-cause mortality and cause-specific mortality. We expect about 150,000 cause-specific deaths determined through VA and about twice as many deaths (300,000) to study all-cause mortality in the 1998–2003 sample frame. The expected age distribution of cause-specific deaths captured with VA, based on the age-specific SRS death rates in 2001, is shown in Figure 2. Using indirect World Health Organization estimates on causes of death in India [1], we also show the approximate numbers of major categories of deaths expected (in thousands) between ages 25 to 69 y (Table 1).

**Table 1:**
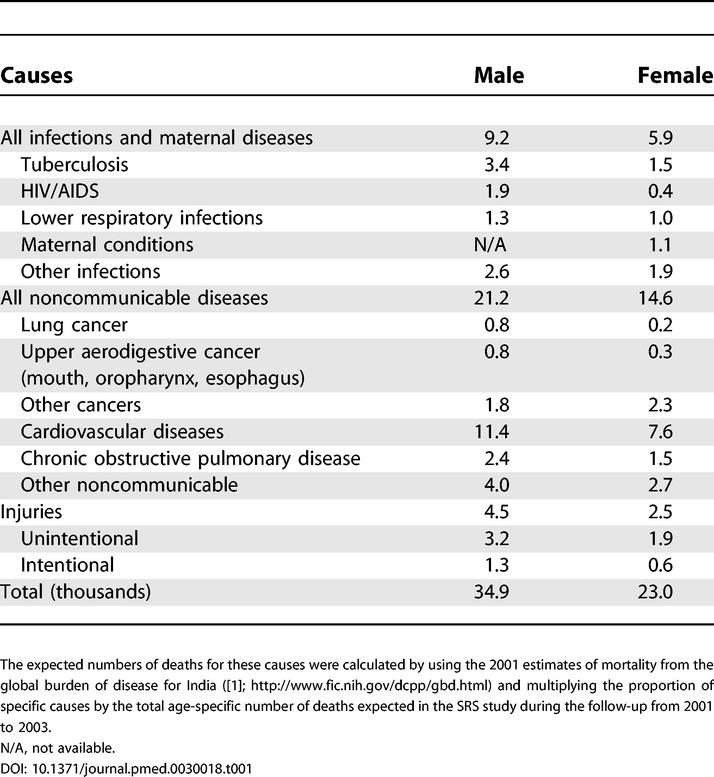
Expected Number of Deaths between Ages 25 and 69 by Cause (Thousands), 2001 to 2003

As of November 2005, 140,000 VA reports have been collected from all SRS units, and about 35,000 records have undergone double physician coding and reconciliation.

Overall we expect several thousand tuberculosis, vascular, and cancer deaths among adults. Such numbers are not excessively large, particularly if the age- and sex-specific relevance of several risk factors is to be assessed simultaneously. For example, assuming a power of 90% and two-sided α of 0.001, and assuming a 40% smoking rate among male controls, the study would have sufficient power to detect relative risks among men as low as 1.4 for lung cancer, 1.1 for all cancers or for tuberculosis, and 1.1 for cardiovascular disease [28]. Thus, the study has robust statistical power to detect small but significant increases in risk for most key variables of interest.

The main planned analyses involve simple tabulations and standard Cox proportional hazards analyses, calculating relative risks that are standardized for age, educational level, and selected covariates as relevant. Analyses of adult deaths will focus on deaths at ages 25–69 y, as these are much less likely to be misclassified than deaths occurring after age 70.

Sample-size estimates for the 2004–2014 sample frame are less certain, as the cause-specific child and adult mortality will depend on the rapidity of declines in childhood mortality and increases in HIV-1-related mortality [11]. However it is reasonable to assume that with expanding sample size (reflecting the growth of populations in the SRS households), some 700,000 deaths will occur over the 10-y period.

### Reliable Measurement of Absolute Rates and Relative Risks

The ability to generate absolute rates depends on completeness of enumeration and being able to calculate underlying demographic denominators, including migration. Evaluations suggest that the SRS has a high ascertainment rate of expected events, although adjustments may be needed for certain age groups. A 1984 study [29] concluded that the system captured 90% of deaths between 1971 and 1980. The SRS, which employs continuous enumeration, is more sensitive at detecting child deaths than are single surveys, and it has recorded more child deaths than those estimated by the National Family Health Survey 2 (NFHS-2), a nationally representative demographic and health survey that interviewed nearly 0.1 million households (http://nfhsindia.org/index.html). Bhat [30] found that adult deaths were underreported by about 13% to 14% (slightly higher in females) and that there is evidence to suggest that the undercount has increased slightly in particular states recently. It is expected that the new SRS sample frame should have corrected the underreporting of adult deaths noted in the first SRS sample frame. Formal demographic evaluation will be done once results from the new SRS are collected. Because household composition is updated every 6 mo, and in-migration and out-migration are traced, it should be possible to calculate absolute mortality rates in the population, including person years at risk. Deaths among in-migrating groups can be excluded, as the death report lists if the person was a usual resident in the SRS unit or not. Some periodic correction for undercount will be needed.

### Loss to Follow-Up

The baseline survey for the study was called the Special Fertility and Mortality Survey (SFMS) and was confined to usual residents of households only [31]. Those living in these households in 1998 were a subselection of the SRS baseline sample frame originally done in 1993. Experience from a prospective study in Chennai (V. Gajalakshmi, personal communication) suggests that if people are resident for a few years, they are not likely to move again. Thus, the enrolled group in the current SRS sample frame is less likely to migrate than would be the general population. This is supported by data from our own small pilot examination of 389 randomly chosen SRS records in two northern states, which showed that only 6% (25/389) of the households had moved from 1998 to 2002.

Nonetheless, we do expect loss to follow-up caused by out-migration from the SRS unit. The risk ratios would be biased downward if such loss to follow-up is nondifferential between exposed and unexposed groups. Similarly, risk ratios would be biased downward if impoverished people (who are most likely exposed to smoking and other risk factors) migrate.

## Results

### Validation Studies of Mortality Outcomes

VA relies on the assumption that most causes of death have distinct symptoms and signs that can be recognized, recalled, and reported by household members or associates of the deceased to a trained, usually nonmedical field-worker. Further, it is assumed that deaths characterized through VA possess a distinct set of features that can be distinguished from other underlying causes of death [32]. Thus, diseases with very distinct symptoms and signs, such as tetanus, that are recognized by the local population may be more suitable for VA than systemic diseases, such as malaria, which is associated with many common symptoms and signs. Factors that influence the validity and reliability of VA include the VA instrument (mortality classification, diagnostic procedures), the data collection procedures (recall period, interviewer's characteristics, respondent's characteristics), and the underlying distribution of cause-specific mortality in a given population [33–35]. Although there is variation in the sensitivity and specificity for specific conditions, VAs are now of established value in helping to classify the broad patterns of mortality for childhood deaths in populations that are not covered by adequate medical services. VAs have also been used to assess the causes of maternal deaths [36].

Background work for this study included two validation studies of adult deaths in India [37–39]. The first study [37,38] developed and tested a VA instrument among 48,000 adult deaths in urban Chennai and 32,000 adult deaths in rural Tamil Nadu, including a 5% random resample. VA conducted by trained nonmedical field-workers resulted in 90% successful reporting on cause of deaths for middle-age adults (25–69 y). The VA instrument reduced the proportion of adult (age 25 or older) deaths attributed to unspecified or unknown causes from 54% to 23% in urban areas and from 41% to 26% in rural areas. VA yielded fewer unspecified causes (only 10%) than the death certificate (37%)–particularly for the deaths that did not occur in hospital–and often yielded somewhat more specific information, for example, about the approximate site of origin of a cancer, or about evidence of tuberculosis, stroke, myocardial infarction, or diabetes (Table 2). The urban Tamil Nadu study also compared VA results to those from a Chennai population-based cancer registry. The VA sensitivity to identify cancer was 95% in the age group 25–69 y, and VA identified 288 deaths that were not registered in the Chennai Cancer Registry.

**Table 2:**
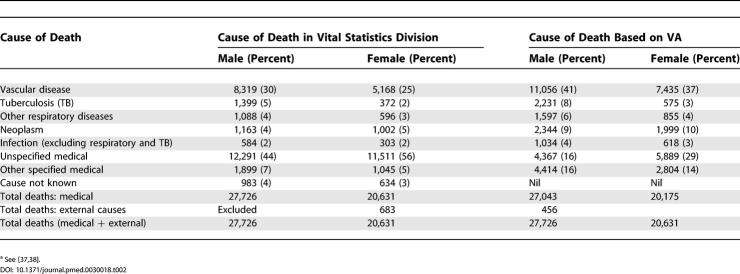
Cause of Death Based on Vital Statistics and VA of 48,000 Adult (>25 y of Age) Deaths in Chennai, India: 1995–1997^a^

The second validation study compared all-cause mortality determined by VA against hospital-based records for 262 adult deaths in northern India [39]. Deaths characterized with VA were compared to medically certified cause-of-death certificates for patients who had died in a hospital. Cause-specific mortality fractions assigned by the VA method were statistically similar to the causes arrived at by review of hospital records (*p* > 0.05). Specificity was high (>95%) for all broad cause groups except cardiovascular (79%) diseases. Sensitivity was highest for injuries (85%), and it was in the range of 60% to 65% for circulatory diseases, neoplasms, and infectious diseases. Sensitivity was low (20% to 40%) for respiratory, digestive, and endocrine diseases. These figures are broadly in agreement with the results from a multicenter validation study of VA for adult deaths conducted in Africa, which found a sensitivity and specificity of 82% and 78%, respectively, for all communicable diseases, and a sensitivity and specificity of 71% and 87%, respectively, for all noncommunicable diseases [34].

Preliminary results from two states indicate that the distribution of underlying causes of death based on the random reinterview does not differ substantially from the cause of death derived from the VA reports of the original RGI surveyors (Table 3).

**Table 3:**
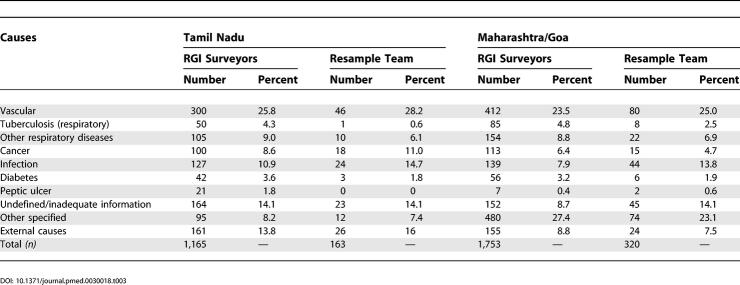
Cause of Death Results for RGI Surveyors and Resample Teams in Tamil Nadu, Maharashtra, and Goa Using VA (Deaths >28 d) in 2003

### Validation Studies of Exposures Measured at Baseline

Baseline exposures were captured in a one-time SFMS conducted within the SRS in February 1998. Baseline exposures captured in the SFMS included socioeconomic information (education, occupation, household income, household composition), water and sanitation facilities and other living conditions, smoking and its type (bidi, cigarette, hukka, or other), age at onset, alcohol use and frequency per week, past history of various medical conditions, and the type of cooking fuel used (major and secondary, and use of a separate kitchen). The survey also recorded deaths, although not their causes, in 1997 [31].

The 2004 baseline survey in the new SRS sample frame (2004–2014) recorded similar exposures and added history of disability from various medical conditions, recent short-term illnesses, tobacco smoking and chewing, alcohol use, vegetarianism, and maternal history, including contraceptive use and pregnancies.

The reliability and representativeness of the SFMS baseline survey can be assessed, in part, by comparing the age-specific prevalence of smoking and alcohol consumption among males found in the SFMS and other standard surveys, such as the Indian Census or NFHS-2. Text S1 provides additional validation studies, such as birth order of children and measures of indoor air pollution.

### Age-Specific Prevalence of Smoking in Adult Males

**Figure 3:**
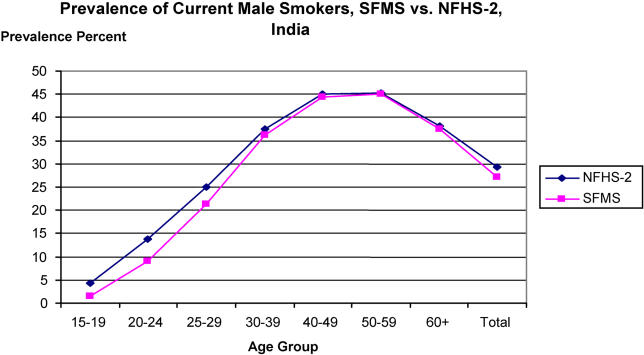
Prevalence of Current Male Smokers: SFMS 1998 versus NFHS-2 1998–1999

Smoking is currently common only among Indian males. The SFMS and NFHS-2 report very similar trends in age-specific prevalence of current male smokers. The steepest increase in prevalence of smoking is between 20–30 y of age (Figure 3). Similarly, the prevalence of smoking among males across 25 states shows a strong correlation between the two studies (Pearson correlation coefficient or *r* ^2^ of 0.92; data not shown). We plan to remeasure the extent of smoking by each of several thousand living adults so as to correct for regression-dilution bias [13]. Additionally, to verify and validate smoking status, we will use a simple hand-held carbon monoxide breathalyzer on about 1,000 smokers and nonsmokers in select states. These simple breathalyzers appear to be effective at detecting smoking status [40].

### Age-Specific Alcohol Consumption in Adult Males

**Figure 4:**
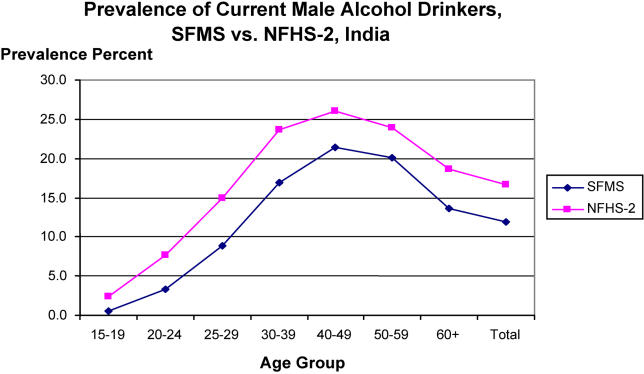
Prevalence of Current Male Alcohol Drinkers: SFMS 1998 versus NFHS-2 1998–1999

As with smoking, a similar pattern in age-specific alcohol consumption is captured by the SFMS and NFHS-2 (Figure 4). The discrepancy in absolute prevalence for each age group may be due to the difference in the sex of the respondents, where females are either overreporting male alcohol consumption in the household in the NFHS-2, or males are underreporting their own consumption in the SFMS. It should also be noted that, unlike the NFHS-2, usually the male head of the household is the respondent of the SFMS.

### Retrospective Household Case-Control and Proportional Mortality Methods

The determinants of death can be identified by comparing risk factors between the dead and living. Such household case-control studies use the dead as cases and their surviving spouses or close relatives as controls. A retrospective study in Chennai of 43,000 male deaths and 35,000 living controls [41], using these methods, has documented that throughout middle age, the death rates from medical causes of smokers were double those of nonsmokers (standardized risk ratio at ages 25–69 of 2.1, with 95% confidence interval 2.0–2.2, smoking-attributable fraction 31%). A large part of this excess risk was from tuberculosis and vascular deaths. If these hazards are similar across India, then about half of all tuberculosis deaths in India could be accounted for by smoking. We will apply similar retrospective methods for smoking, tobacco chewing, and alcohol, as these exposures are gathered for dead adults and from a living household respondent.

Simply asking about the dead person's risk factors could be useful. A recent retrospective study of 1 million deaths in China compared the proportions of smokers and nonsmokers who have died of tobacco-attributable diseases versus nontobacco-related diseases (chiefly injuries), to calculate the excess in smokers [42]. Our preliminary pilot studies among childhood deaths in Northern India found that 61% of children (16/26) who died of vaccine-preventable diseases were not immunized in comparison to 40% of control children (18/45) who died from injuries. The crude odds ratio of 2.4 suggests that half of the vaccine-preventable child deaths need not have occurred.

Using these two retrospective methods, we are conducting validation studies of selected risk factors such as childhood immunization, childhood malnutrition, alcohol, male time away from home (as a proxy of HIV-1-related sexual risk taking), and other variables.

## Discussion

### Key Design Challenges

Several challenges stand out in the design and implementation of this massive study. First is its sheer size. A total of 14 million people is a large sample frame, but commensurate with the needs of monitoring health status among the one billion people in India. The study builds upon the routine infrastructure that has been in place for over 30 y within the SRS. It works with the leading demographic research organization in India (for example, the decennial census involves the RGI hiring two million enumerators to survey about 150 million households within 25 d). Second is the need for simplification of methods to make such a scale of study practicable. The study is not the only epidemiological study needed in India, but its unique focus and scale raise important challenges in the development of new methods. The Million Death Study uses a “large simple” design, which places specific demands on ensuring that new procedures (such as the new VA instrument and physical and biological measurements) are rigorously tested, piloted, and simplified. The third challenge is sustainable funding. The current SRS sample frame is being followed on a total incremental budget of US$2 million (or less than 33¢ per person). This does not include the core funding of the RGI surveyors and related infrastructure. With inclusion of those, the overall study can still be done in a highly cost-effective manner at well under US$1 per person. Collection, processing, and long-term storage of biological samples is expected to cost US$2–5 per person for dried blood spots and US$10–20 per person for a 10-ml tube of blood. These costs compare extremely favorably with those existing biobanks in the United Kingdom and elsewhere [43,44]. Most of the infrastructure costs will be sustained by the Government of India. However, we believe that redirecting some of the considerable, and often inefficient, spending on monitoring individual disease projects is required to enhance the SRS.

### Key Design Issues for Blood-Based Genetic Epidemiology

Discovery of “new” risk factors should benefit from the recent and extraordinarily rapid progress by many different research groups and biotechnology companies in developing low-cost, miniaturized methods for the simultaneous assay in small volumes of blood (or, perhaps, dried blood spots) of vast numbers of nucleic acid fragments, host genetic factors, proteins, small molecules, and pathogens [45]. This rapid biotechnological progress has been backed by increasingly sophisticated computer software to help interpret the mass of numerical information that can be generated from each person's blood, yielding within the next few years many, as yet unforeseen, qualitatively different analytic capacities. Appropriately large-scale epidemiological studies that acquire blood (or other) samples from individuals and systematically link them to relevant measures of disability and future mortality are required to make such technological progress relevant to human populations. It is particularly important for such studies to address the shortcomings of the existing biological sample collections that are now being undertaken [43,44], including the particular circumstances of India and the specific infectious diseases common to developing countries.

Key issues which arise in moving to blood-based epidemiology include the choice of blood sample, long-term storage and retrieval, and statistical design issues.

### Choice of Blood Sample

Biological specimen collection procedures will build on experience in China, India, and elsewhere. We will undertake pilot studies to test the feasibility and acceptability of methods, to ensure that biological samples collected can serve as a long-term source of DNA for genotyping and that material collected is suitable for biochemical, hematological, proteomic, and other assays [46]. Major options include a nonfasting blood sample collected into one 10-ml ethylenediaminetetraacetic acid Vacutainer, which has been shown previously by the University of Oxford laboratory to allow a wide range of assays [47]. This system is used by the ongoing Chinese Kadoorie Study of 500,000 adults and by the UK Biobank project of the same size [43]. Alternatively, dried blood samples on filter paper have the advantages of easy storage and transport, as well as being less intrusive (i.e., by finger prick rather than by venipuncture). Dried blood spots have been recommended by the World Health Organization for use in field HIV-1 investigations and have been used in various studies within India [48,49].

We currently plan pilot studies of dried blood spots or tubes of blood among 4,000–9,000 adults in several SRS units in four to five states. These pilots will focus on standardizing methods to obtain anthropometric, behavioral, and physiologic measurements and to evaluate the alternative methods for collection of biological specimens for their utility, cost, and practicability (dried blood spot or tube of blood). The pilots will focus on feasibility of field methods, simplification of approaches, and quality control. The results of the pilot studies, which are expected by June 2006, will help inform the design of the larger survey. A special survey will help define practicable questions on HIV-1 risk behaviors.

### Long-Term Storage, Retrieval, and Analyses

We are undertaking systematic reviews of the literature and reviewing available assays in India to examine which current bioanalytic methods can be used to test current hypotheses (either for correlates of infection or chronic disease). Using the above pilot samples, we will develop testing and analytic methods for biological samples that will permit high-throughput, low-cost, and high-quality assays to be run and also permit the long-term reliable storage and retrieval of such samples. Dried blood spots may well be stable in a basic 4 °C refrigerator, with minimal storage requirements [46]. With a population of one billion, India certainly requires at least two major biorepositories (including splitting samples, which safeguards against loss at one facility). We are developing plans for long-term biorepositories with the ability to store samples for decades. Much will depend on the choice of the final assay.

### Nested Case-Control Methods and Genetic Association Studies

There is a long list of biological factors in blood that might be correlates of disease-specific mortality. To be efficient, we will use a nested case-control approach in our prospective study. Biological samples are taken from all adults in a baseline survey and stored long-term; then, when sufficient numbers of cases with the disease of interest have died (based on the 6-mo follow-up of the causes of death), aliquots from those cases are retrieved from storage, plus aliquots from a few matched controls per case; and, finally, the factors of interest are assayed in these cases and these controls. This design is similar to that in the Chinese Kadoorie Study and the UK Biobank project [43]. As noted above, the number of deaths is likely to be substantial for most of the common diseases of interest to make this an efficient strategy.

Under some circumstances, studies that used population-based controls to study gene-disease associations have been biased due to underlying variation in gene frequencies between populations (“population stratification” [50]). Genetic epidemiology can provide reliable population-based estimates of disease–allele frequency, penetrance, and attributable risk, particularly if designs that account for this bias are employed, and accordingly this has led to greater emphasis recently on family-based association designs. However, there is also evidence that well-designed population-based studies are sometimes superior [51]. The SRS offers a unique opportunity to explore statistical design issues, as both general population and family-based sampling is possible. Family-based studies allow for population-specific estimates of clustering of disease and correlation (heritability). Further, families with a large number of siblings could be oversampled to increase power for genetic linkage studies. The use of “genomic controls” where anonymous markers are genotyped to test for population stratification can be employed [52].

### Limitations

The study has several limitations. First, despite a very large sample size, the statistical power remains modest for less common causes such as those leading to maternal deaths. However, for most of the major public health conditions of importance, sufficient events should occur to generate plausible absolute rates and relative risks for risk factors. Second, careful attention in design means that most identified biases should be minimized and should ensure high internal consistency of fieldwork and coding. However, periodic revalidation of mortality outcomes against external standards will be needed. Similarly, continuous improvement of exposure measurements, partially through careful pilots, will be needed. Third, VA yields broad classification of the underlying causes in about 90% of deaths before age 70. In old age, however, the proportion of classifiable deaths is lower. For some specific conditions, such as childhood pneumonia, the cause-specific fractions may be difficult to estimate below certain levels [53].

### Significance to Global Health

The Million Death Study will reliably document not only the underlying cause of child and adult deaths, but also key risk factors (behavioral, physical, environmental, and eventually, genetic). It offers a globally replicable model for reliably estimating cause-specific mortality, using VA, and strengthens India's flagship mortality monitoring system. Despite the misclassification that is still expected, the new cause-of-death data will be substantially better than that available previously in India. The study builds India's capacity for research and for public health action. It provides a large, representative, low-cost and long-term system to reliably track the health status of one billion people for the next decade or longer.

## Supporting Information

Text S1 Supplemental Text October 14 (80 KB DOC).

Text S2 Ethics Approval ICMR (132 KB JPG).

Text S3 Ethics Approval PGI (117 KB JPG).

**Patient Summary**

**Background**

A few pieces of information are crucial to measure the health status of a population, which permits a sound basis for health policy. One basic question is which diseases people die from. To answer it, one needs to know the numbers and causes of death. The age at which people die is critical to estimate the burden for society and to develop strategies to prevent some of the deaths. In developed countries, doctors determine and certify the causes of most deaths that occur in hospitals. However, the majority of deaths worldwide in developing countries occur at home and without reliable recording of their causes and distribution. The present study takes place in India. About one in six deaths worldwide occurs in India, or about 9.5 million deaths per year. Only about a third of these deaths are registered.

**Why Is This Study Being Done?**

The Indian government recognized the need for better data on the distribution and causes of deaths across the country and, together with an international group of researchers, is undertaking a large study over 16 years (from 1998 to 2014) to collect representative data for the country.

**What Are the Researchers Doing?**

In this article, the researchers who came up with the plan for the survey and will oversee its implementation describe and discuss the details of the project. They will monitor, through regular visits and interviews by surveyors, the health status of nearly 14 million people in 2.4 million representative households from all over India. The surveyors, who will be trained for the job but not have a medical background, will collect information about the health status of all household members and about key risk factors for disease such as smoking, alcohol use, childhood immunization, and indoor air pollution. About one million deaths are expected to occur among these people in the study period. As part of their interviews, the surveyors will conduct “verbal autopsies,” that is, ask specific questions about how someone died, to determine the causes of death. Each verbal autopsy will be checked by two independent physicians (and a third if there is disagreement) before the cause of death is recorded. The researchers also describe their plans to extend the survey–they plan to collect simple physical measurements such as blood pressure, height and weight, and blood samples (most likely through a simple spot of blood taken from the finger) from the participants. These biological samples contain information about the genetic makeup of the individuals and, potentially, on other markers that might be connected to the cause of death, such as exposure to environmental toxins, the presence of viral or bacterial infections, and others. This information will help them to understand what caused the diseases that the people died from.

**What Does This Mean?**

This study will provide the most accurate picture of causes of childhood and adult death in India, as well as document key risk factors. By studying diseases that are common in one part of India but not in another, new risk factors will be identified and can be used to reduce deaths overall. It will also serve as an example for other countries.

**Where Can I Find More Information Online?**

The following Web sites provide relevant information on this and similar large studies.

Full protocol, field instruments, training manuals, and more details for this study:

http://www.cghr.org/project.htm

Home page of the UK Biobank:

http://www.ukbiobank.ac.uk/

Kadoorie study of chronic disease in China:

http://www.ctsu.ox.ac.uk/~kadoorie/public/

The World Health Organization's Health Metrics Network:

http://www.who.int/healthmetrics/en/

Indepth Network for Continuous Demographic Evaluation of Populations and Their Health in Developing Countries:

http://www.indepth-network.net

## Abbreviations

ICD-10: *International Statistical Classification of Diseases and Related Health Problems*
NFHS-2: National Family Health Survey 2
RGI: Registrar General of India
SFMS: Special Fertility and Mortality Survey
SRS: Sample Registration System
VA: verbal autopsy
