# Supplementary material for: Prospective Study of One Million Deaths in India: Rationale, Design, and Validation Results
Source: PLoS Med. 2005 Dec 20;3(2):e18. doi: 10.1371/journal.pmed.0030018 (PMC1316066; doi:10.1371/journal.pmed.0030018)
Supplement: Supplementary file 1 [file pmed.0030018.st001.doc]

## **Supplemental Text: Additional details of field collection methods, study organization and validation experience to date**

## **I. Field Methods**

## Data collection with the VA instrument

VA forms were designed after seeking expert opinion from the World Health Organization (WHO; (1)), and after review of the literature on validation studies (1-4). The methodology of writing the VA report is based on the large-scale studies done in Tamil Nadu (4;5). The first VA forms were extensively piloted in five states in April 2002, and their results were reviewed by an expert panel of WHO scientists in June 2002. The early review found that even basic training (one day) decreased the proportion of deaths for which there was only a one-word narrative from 50% to 5%. With more substantial training, the RGI surveyors were able to raise the proportion of deaths for which a cause is available from about 50% to over 85% of deaths before age 70.

Based on these pilots and review, we decided to adopt and develop an open/closed format (forms are found at [www.cghr.org/project.htm](http://www.cghr.org/project.htm)). We conducted a small pilot of 262 adult deaths and found that if the open-ended narrative alone is used, trained physician coders were able to assign an underlying cause of death for 56% of the records (6). If the questions alone are provided, then trained physician coders were able to assign an underlying cause of death for only 49% of the VA records. When the narrative and questions were provided together, the proportion of classifiable VA records rose to 65%. Our results suggest that high quality narratives are the single most important factor in increasing the proportion of classifiable causes of death.

In the closed section, “filter” questions are used to elicit the presence or absence of specific signs and symptoms. If the filter question is positive, then subsequent questions are provided to determine the severity, duration, or other characteristics of these symptoms. For adult deaths, there is a list of signs and symptoms used by the RGI surveyor to obtain more detailed information about the cause of death. The symptom list is used as a filter to define additional probing questions that should be asked if the respondent mentions a particular symptom during the verbatim account of the death. RGI surveyors go through each symptom to ensure that key filter symptoms have not been missed. The written narrative details the following information: associated signs and symptoms in chronological order; duration; sudden or gradual onset of illness; type of treatment if any treatment received; details on hospitalization prior to death; name and location of hospital; duration of hospitalization; history of similar episodes and treatment given; and abstract information related to the illness prior to death from available investigation reports, death certificate, or discharge summaries. Each interview lasts about 30-45 minutes.

Among the first few thousand deaths, the proportion of VA records coded with high certainty of diagnosis does not vary according to relationship of respondent to the head of the household (family relative, other relatives or neighbor; data not shown). Whether or not the respondent lived with the deceased during the illness that lead to death is the more important determinant of obtaining classifiable causes.

All forms have a common format that includes a socioeconomic and demographic profile of the respondent and the deceased, details of the illness and a narrative section. Forms are available in English or Hindi, with the narrative written in the local language. Our current VA instrument is in its seventh generation and is provided as a single-page, double-sided layout in easy-to-carry booklets with simple instructions for their use. Each type of form (neonatal, child, adult, and maternal) is color-coded and bound according to the type of form for ease of identification in the field.

## Random re-sample of RGI surveys

To ensure high quality fieldwork, a specialist re-sample team directly reporting to the study investigators re-interviews 10% (randomly chosen) of households. These are also submitted for central medical review and revalidated. During the early phase of the study, about 10% of each RGI surveyor’s visits will be re-sampled, so as to provide early training input and correction of methods. During the later phases, the percentage will be reduced to about 5%. On average, each state will have about two re-sample team members, each covering about one unit per RGI surveyor (i.e., about 1 out of the 10-12 units sampled by a RGI surveyor), or about 15 units per re-sample team. RGI surveyors will receive feedback on the completeness of their work from the work of the re-sample team. As noted in the main text (table 2), the correlation between the random audit team and the RGI supervisors on overall distribution of causes of death was high.

## Double coding by trained physicians

Previous validation results of VA for adult deaths suggested that central diagnosis by a trained panel of physician coders yielded consistently higher sensitivity for the cause of most specific mortality outcomes than opinion-based algorithms (7). For child deaths, physician coding is comparable or better than algorithms (8). In this study, in order to reduce inter-observer variation, two trained physician coders independently examine each VA report and determine a probable underlying cause of death coded in the International Classification of Diseases-10^th^ revision (ICD-10; (9)). Before assigning cause of death, the physician coders are trained to carefully screen all relevant information provided, noting all of the positive evidence, and use clinical judgment in assigning the underlying cause of death. For each VA record, physician coders will provide the following information: an underlying cause of death in words (e.g., “tuberculosis”); corresponding ICD-10 code (e.g, A15); and the key words used to guide and support their decision. If two physicians do not agree on an underlying cause of death, a web-based system assigns to each physician the original report and the ICD-10 code of the other physician (without revealing the identity of the other physician). The physician coders are then required to use the additional information provided by the third physician (ICD-10 and key words) to reach an agreement on underlying cause of death. An expert panel of senior physician coders will review VA records where two physicians cannot agree on a cause of death after one reconciliation attempt. Physicians are drawn from across India, so as to ensure that cross-state comparisons are valid.

A pilot of double coding of 1,198 VA records was conducted in the Karnataka state. In 84% of the cases, two independent physicians were able to code to a common cause after only one round of VA training. We expect approximately half of the outstanding differences to be “minor” differences that should be easily resolved after a reconciliation attempt, thus yielding about 85 to 90% first-round agreements between two independent physician coders.

*Pilot studies of physical, behavioral and biological measurements*

Physical measurements for adults will involve blood pressure, height, weight, waist/hip circumference, and lung function. From children, simple height and weight data will be collected. We place special emphasis on obtaining measures of adult obesity, especially as the patterns differ greatly from developed countries. Several of our collaborators have implemented simple physical measurements in over 700,000 adults (including prospective studies numbering 550,000 adults in Chennai, Tamil Nadu; 150,000 adults in Mumbai, Maharashtra, and 100,000 adults in Trivandarum, Kerala), and shown that non-medical staff can reliably obtain simple physical measurements. One study (10;11) has surveyed over 100,000 adults in Mumbai and found elevated body mass index [or BMI greater than 25 kg/m2] in approximately 30% of men and women over age 35 and low BMI [<18.5 kg/m2] in some 20% of adults. Thinness was common among illiterate men, and was associated with smoking and chewing tobacco, whereas higher education was associated with raised BMI. The same study has also reported on blood pressure among nearly 89,000 adults (12).

Behavioral pilot studies will focus on HIV-1 risk taking such as sexual partnerships outside marriage. However, such self-reported behavior is greatly misreported (13) and past surveys in India have often had low participation rates. Thus careful pilots will be undertaken prior to a larger study of 5,000 adults in one state. Additional surveys of high-risk populations within SRS areas will also be done to understand spread of HIV-1 and of risk behavior.

## Data management

Previous versions of the paper-based VA form were entered (about 40,000 records) in four regional centres in India using Microsoft Access. The written narrative was scanned and retained as a linked image file. A 100% re-check of printouts verified entries. For the most recent round of VA fieldwork (about 110,000 deaths completed in March 2005) and for future entries, central data entry will be performed using scannable, double-sided optical readers. A 100% on-screen re-check of all fields will be done. The written narrative will be retained as an image file for permanent storage and to facilitate re-checks and sub-studies. All VA records are then compiled into a MySQL database for data verification and cleaning.

After scanning, select information (removing personal identifiers) and a complete image of the VA narrative are extracted from the VA database for each record. These fields and an image of the narrative are used to create modified VA reports, entitled “Physician Reports”. Custom-designed, internet-based software permits the electronic distribution and management of physical reports, as well as the remote collection of cause of death information. Briefly, this system creates Physician Reports from the consolidated VA database, assigns Physician Reports to the appropriate physician (based on language of the narrative and the physician’s VA workload), captures the underlying cause of death information, and manages and monitors the administrative tasks related to cause of death coding. This web-based system allows centralized management of the distribution of all VA records, and the secure collection of cause of death data from all parts of India.

*Data linkage*

In order to better understand the risks for death, each cause-specific death will be linked to its respective baseline record. For the first SRS sample frame, events are linked to the computerized Special Fertility and Mortality Survey (SFMS) of February 1998. For the new SRS sample frame, the linkage will be with the computerized 2004 baseline survey (see below for a list of exposures recorded in each). Additional special surveys will be introduced into the new SRS sample frame for follow up. The new SRS has introduced a unique 9-digit identification number for each individual that can reliably track in-migration, out-migration, vital events and family additions (such as births).

The first SRS sample frame had no such unique identification number. Thus, matching will be based on other variables. Each unique SRS unit is limited to about 150 households. Therefore, matching based on household number, relationship to head of household, gender and name within each of the SRS units can potentially yield a high degree of matching. However, if household numbers are incorrectly assigned in the baseline survey, then manual matching of paper-based forms is likely to be required. A pilot of 389 paper-based SRS death records matched to the SFMS demonstrated that if a paper-based SFMS record could be identified with proper linkage to the death record through the SRS unit number and household number, then matching was successful for 84% of deaths. In-migration (particularly of elderly mothers) and out-migration accounted for an equal proportion of records (about 7% each) that were not successfully matched. Our efforts at data linkage of electronic records have been less successful primarily as a result of variability in the recording of the household number(s) in the paper-based SRS records. We are currently addressing this issue by extracting and linking select variables (such as name) from several SRS forms or schedules, which will enable us to correctly identify SFMS records for most deaths that occur within the SRS sampling frame.

## Training of field interviewers and physician coders

Most RGI surveyors are male with at least 12 years of formal (non-medical) education. All RGI surveyors and re-sample teams undergo a standardized, six-day training session in VA methodology. This training is composed of two days of in-class instruction followed by four days of fieldwork, discussion and feedback. VA training includes an introduction to human anatomy and the signs and symptoms of common diseases, mock field interviews with methods to canvas each VA question, hands-on VA writing, and a feedback session to evaluate and improve the training methods. The training aims to improve the surveyors’ ability to collect data from the respondents in the open/closed format using symptom checklists and probing questions. The goal is to obtain a complete and logical history of the signs, symptoms and supportive details of each death. The surveyors are trained to seek information from the person with the most details of the illnesses and symptoms prior to death (for all medical causes). For example, for child and neonatal deaths, mothers should be the principle respondent. Each surveyor is required to complete a number of mock VA reports using the techniques and materials provided during the training. Training in VA will be provided as part of the routine activities included within the new SRS sampling frame, and repeated prior to each half-yearly survey. Over 800 RGI surveyors and senior staff have received at least two rounds of training in VA from December 2002 to December 2004.

A network of 15 academic partners from various states work in collaboration with the RGI and the WHO to ensure standardized training, random re-sampling, and to build skills for sustainable mortality measurement.

All VA physicians undergo multiple rounds of training in cause of death assignment. This three-day training covers the importance of VA and how it works, orientation to ICD-10, hands-on exercises in VA, individual work on VA reports in the local language with group discussions on challenging cases, and post-test evaluations/feedback. All physicians have access to web-based training tools, including case-studies, diagnostic guidelines and ICD-10 lists. Senior physician coders review the first 50-100 reports of all new physician coders. The modest honorariums for physicians are paid only on completion of all steps, including reconciliation with another physician. We anticipate training about 150-200 physicians for a cause of death coding panel, depending on language requirements across the world. As of May 2005, over 95 physicians have been trained.

## Research Ethics

SRS enrolment is on a voluntary basis, and its confidentiality and consent procedures are defined as part of the Registration of Births and Deaths Act, 1969. Oral consent was obtained in the first SRS sample frame. The new SRS sample obtains written consent at the baseline. Families are free to withdraw from the study, but the compliance is close to 100%. The study poses no or minimal risks to enrolled subjects. All personal identifiers present in the raw data are anonymized before analysis. The study has been approved by the review boards of the Post-Graduate Institute of Medical Education and Research, the Indian Council of Medical Research, and the Health Ministry’s Screening Committee. Specific written consent procedures for additional biological measurements will be added, using international guidelines (14,15).

**II.	Study organization and study schedule**

The study is implemented by a large interdisciplinary team. The RGI-CGHR office in Delhi is responsible for day-to-day management, coordination with states and government offices, and centralized data entry. A Global Coordinating Centre is at the University of Toronto. This Centre provides overall strategic guidance, quality control, and manages the web-based physician coding system. Fifteen Academic Partners in the major Indian states (see list in main report) are responsible for training, coding, and re-sampling in their home states. A national advisory committee comprised of the RGI and project investigators and co-chaired by Professors Vendhan Gajalakshmi and Rajesh Kumar provides input into the project progress.

*Independence of the SRS*

The SRS is managed by the Office of the Registrar General within the Ministry of Home Affairs of the Government of India. Notably, the Registrar General operates independently of the Ministry of Health and Family Welfare or any disease control programs. This permits *de facto* separation of the producers and potential users of vital registration data.

*Role of funding agencies*

External funders have no role in study design, data collection, data analysis, data interpretation or writing of publications.

*Study schedule*

The timetable for the study involves producing two major reports and publications on the baseline characteristics of 2.4 million homes (1.1 million homes in the 1998 SFMS, and 1.3 million homes in the 2004 baseline survey for the New SRS) in 2005. Preliminary cause of death data on about 40,000 deaths at the national level should be available by December 2005, depending on progress in physician coding. The remaining causes of death from the first SRS sample frame will be double coded and analyzed by mid 2006, as will the first 60,000 or so deaths in the new SRS sample frame. Data linkage efforts to link the 1998 SFMS with the deaths from 1998-2003 will be completed by December 2007. Pilot studies of physical and biological measurements surveys within the SRS will begin in fall 2005.

**III.	Additional validation studies of exposures**

Birth order: The percentage of births in the year of the survey that were first, second, third or fourth or higher order births were compared in the bigger states (Assam, Andhra Pradesh, Bihar, Gujarat, Haryana, Himachal Pradesh, Karnataka, Kerala, Madhya Pradesh, Maharashtra, Orissa, Punjab, Rajasthan, Tamil Nadu, Uttar Pradesh and West Bengal) between the SFMS and the NFHS-2. Pearson correlations (R^2^) across states were 0.94, 0.89, 0.49 and 0.94 for the first, second, third and fourth births, respectively (data not shown).

Indoor air pollution: The use of “dirty fuels” (firewood, dung and crop residue) has been measured as an indicator of indoor air pollution in households in both the SFMS and the 2001 Census. A comparison of the proportion of households that use such dirty fuels for the bigger Indian states reveals a high correlation (R^2^ = 0.8) between the two surveys (data not shown).

Reference List

(1)	Anker M, Black R, Coldham C, Kalter H, Quigley M, et al. (1999) A standard verbal autopsy for investigating causes of death in infants and children. WHO; Report No.: WHO/CDS/CRS/ISR/99.4.

(2)	Quigley MA, Chandramohan D, Rodrigues LC (1999) Diagnostic accuracy of physician review, expert algorithms and data-derived algorithms in adult verbal autopsies. Int J Epidemiol 28:1081-1087.

(3)	Kumar R, Sharma AK, Barik S, Kumar V (1989) Maternal mortality inquiry in a rural community of north India. Int J Gynaecol Obstet 1989 29: 313-319.

(4)	Gajalakshmi V, Richard P, Santhanakrishnan K, Sivagurunathan B (2002) Verbal autopsy of 48,000 adult deaths attributed to medical causes in Chennai (formerly Madras), India. BMC Public Health. 2:7.

(5)	Gajalakshmi V, Peto R (2004) Verbal autopsy of 80,000 adult deaths in Tamil Nadu, South India. BMC Public Health 4: 47.

(6)	Kumar R, Thakur J, Rao M, Singh M, Bhatia P (2005) Validity of Verbal Autopsy in Determining Causes of Adult Deaths. Indian Journal of Public Health. In press.

(7)	Chandramohan D, Maude GH, Rodrigues LC, Hayes RJ (1988) Verbal autopsies for adult deaths: their development and validation in a multicentre study. Trop Med Int Health 3:436-446.

(8)	Kalter HD, Gray RH, Black RE, Gultiano SA (1991) Validation of the diagnosis of childhood morbidity using maternal health interviews. Int J Epidemiol 20:193-198.

(9)	WHO (2003) Manual of International Classification of Diseases, Injuries and Causes of Death (Tenth revision). Geneva, Switzerland: World Health Organization.

(10)	Gupta PC, Mehta HC (2000) Cohort study of all-cause mortality among tobacco users in Mumbai, India. Bull World Health Organ 78: 877-883.

(11)	Shukla HC, Gupta PC, Mehta HC, Hebert JR (2002) Descriptive epidemiology of body mass index of an urban adult population in western India. J Epidemiol Community Health 56: 876-880.

(12)	Gupta PC, Gupta R, Pednekar MS (2004) Hypertension prevalence and blood pressure trends in 88,653 subjects in Mumbai, India. J Hum Hypertens 18: 907-910.

(13)	Cleland J. Boerma JT, Carael M, Weir SS (2004) Monitoring sexual behaviour in general populations: a synthesis of lessons of the past decade. Sex Transm Infect. 80: Suppl 2 1-7.

(14)	Government of India (1998) Revised Guidelines for Exchange of Human Biological Material for Biomedical Research Purposes. Indian Journal of Pharmacology 30: 56-57.

(15) [Clayton EW, Steinberg KK, Khoury MJ, Thomson E, Andrews L, Kahn MJ, Kopelman LM, Weiss JO.](http://www.ncbi.nlm.nih.gov/entrez/query.fcgi?cmd=Retrieve&db=pubmed&dopt=Abstract&list_uids=7500511&query_hl=32) (1995) Informed consent for genetic research on stored tissue samples. JAMA. 274: 1786-1792.
